# Fibrinogen and associated risk factors in a high-risk population: urban indigenous australians, the druid Study

**DOI:** 10.1186/1475-2840-9-69

**Published:** 2010-10-29

**Authors:** Louise J Maple-Brown, Joan Cunningham, Nirjhar Nandi, Allison Hodge, Kerin O'Dea

**Affiliations:** 1 Menzies School of Health Research, Charles Darwin University, Darwin, Australia; 2 Division of Medicine, Royal Darwin Hospital, Darwin, NT, Australia; 3 Department of Medicine (University of Melbourne), St. Vincent's Hospital, Melbourne, Australia; 4 Sansom Institute for Health Research, UniSA, Adelaide, Australia

## Abstract

**Background:**

Epidemiological evidence suggests that fibrinogen and CRP are associated with coronary heart disease risk. High CRP in Indigenous Australians has been reported in previous studies including our 'Diabetes and Related diseases in Urban Indigenous population in Darwin region' (DRUID) Study. We studied levels of fibrinogen and its cross-sectional relationship with traditional and non-traditional cardiovascular risk factors in an urban Indigenous Australian cohort.

**Methods:**

Fibrinogen data were available from 287 males and 628 females (aged ≥ 15 years) from the DRUID study. Analysis was performed for associations with the following risk factors: diabetes, HbA1c, age, BMI, waist circumference, waist-hip ratio, total cholesterol, triglyceride, HDL cholesterol, C-reactive protein, homocysteine, blood pressure, heart rate, urine ACR, smoking status, alcohol abstinence.

**Results:**

Fibrinogen generally increased with age in both genders; levels by age group were higher than those previously reported in other populations, including Native Americans. Fibrinogen was higher in those with than without diabetes (4.24 vs 3.56 g/L, p < 0.001). After adjusting for age and sex, the following were significantly associated with fibrinogen: BMI, waist, waist-hip ratio, systolic blood pressure, heart rate, fasting triglycerides, HDL cholesterol, HbA1c, CRP, ACR and alcohol abstinence. On multivariate regression (age and sex-adjusted) CRP and HbA1c were significant independent predictors of fibrinogen, explaining 27% of its variance; CRP alone explained 25% of fibrinogen variance. On factor analysis, both CRP and fibrinogen clustered with obesity in women (this factor explained 20% of variance); but in men, CRP clustered with obesity (factor explained 18% of variance) whilst fibrinogen clustered with HbA1c and urine ACR (factor explained 13% of variance).

**Conclusions:**

Fibrinogen is associated with traditional and non-traditional cardiovascular risk factors in this urban Indigenous cohort and may be a useful biomarker of CVD in this high-risk population. The apparent different associations of fibrinogen with cardiovascular disease risk markers in men and women should be explored further.

## Background

Indigenous Australians have rates of cardiovascular disease (CVD) mortality some 7-10 times higher than non-Indigenous Australians at ages 25-64 years and a life expectancy that is 15-20 years shorter [1]. Fibrinogen is an inflammatory biomarker that has been independently associated with CVD outcomes (both coronary heart disease and stroke) [2]. Fibrinogen plays a key role in thrombus formation, platelet aggregation and is a major contributor to plasma viscosity. It is also an acute phase reactant, increased in inflammatory states, and is closely associated with C-reactive protein [3]. As expected, fibrinogen is associated with traditional cardiovascular risk factors such as age, cigarette smoking, lipids, body mass index, diabetes, and blood pressure in many population groups (including North American whites, Japanese and Native Americans) [3-6]. Fibrinogen has recently been associated with impaired myocardial systolic function [7] and abnormalities of circadian blood pressure variability and endothelial function in obese participants [8]. Higher fibrinogen has also been associated with alcohol abstinence and socio-economic disadvantage [3,9].

Indigenous Australians (Aborigines and Torres Strait Islanders) in remote areas have been reported to have high fibrinogen levels compared to other population groups; fibrinogen increased with age and was associated with traditional cardiovascular risk factors but these factors only explained 12% of the variance in fibrinogen on multiple regression analysis [10]. Similarly, fibrinogen levels have been reported to be high in other Indigenous populations internationally, including Canadian Aboriginals and Native Americans [11,12]. In the Strong Heart Study of Native Americans, fibrinogen levels predicted cardiovascular events independent of traditional risk factors in a population-based sample of adults without clinical evidence of coronary heart disease at baseline [6]. The addition of fibrinogen (along with urine albumin-creatinine ratio, ACR) to standard clinical and laboratory risk factors improved discrimination of a multimarker model for prediction of CVD events [12].

Most studies of Indigenous Australians concern those living in rural and remote regions. Less is known about health patterns in urban areas, where the majority of Indigenous Australians live [13]. The Darwin Region Urban Indigenous Diabetes (DRUID) Study was designed to address this knowledge gap. The aims of this paper are to evaluate levels of fibrinogen and its association with traditional and novel cardiovascular risk markers in an urban Indigenous Australian cohort, a population with high rates of diabetes, obesity and premature CVD.

## Methods

### Participants

The DRUID Study was a cross-sectional study of approximately 1,000 urban Indigenous people from Darwin, Australia, undertaken from September 2003 to March 2005. Darwin, the capital of the Northern Territory, is the northern-most capital city in Australia and is situated on the Timor Sea, thus is relatively close to Asia. In 2001, Indigenous people represented approximately 2% of the Australian population, 29% of the Northern Territory population, and 10% of the Darwin Region population [13,14]. DRUID participants met the following eligibility criteria: self-identified as Aboriginal and/or Torres Strait Islander; aged ≥ 15 years; had resided within a specified geographical region around Darwin for at least six months; and living in a private dwelling. All participants underwent a 75 gm oral glucose tolerance test (OGTT) unless pregnant or on medications for diabetes. Blood samples were taken fasting and at 2 hours (for those who had OGTT). The DRUID study was a volunteer cohort including approximately 14% of the estimated target population, and therefore not necessarily representative of the target population. The population, methods and response rates have been previously described [15].

### Anthropometry, blood pressure, biochemistry and health behaviours

Measures were performed as described previously [15,16]. In brief: body weight was recorded to the nearest 0.1 kilogram using a digital scale weighing up to 200 kg (Model 767, Seca Deutschland, Hamburg, Germany); height was recorded to the nearest 0.1 cm using a portable stadiometer (Model PE87, Mentone Educational Centre, Moorabbin, Victoria, Australia); waist and hip circumferences were measured to the nearest 0.1 cm using a 2-metre non-stretch fiberglass tape; sitting blood pressure and heart rate were measured using a Welch Allyn Spot Vital Signs monitor (Welch Allyn Medical products, Skaneateles Falls, USA). Smoking status and alcohol abstinence were based on self-report. The following parameters were measured in fasting blood as previously described [15]: HbA1c (EDTA whole blood); glucose, insulin, homocysteine (fluoride/EDTA plasma); cholesterol, triglycerides, HDL cholesterol, hs-CRP (serum). Albumin and creatinine concentrations were measured in urine as previously reported [15]. Diabetes diagnosis was based on OGTT, using the 1999 WHO diabetes classification [17].

Fibrinogen was measured by Clauss method on IL Futura Plus by the Clinical Trials Laboratory (Flinders Medical Centre, Bedford Park, SA) [15]. A total of 1,004 participants provided at least 1 measurement in DRUID; n = 89 were excluded from this analysis as they did not have a blood sample for assessment of fibrinogen level, thus n = 915 were included in this analysis.

### Statistical Analysis

Data analysis was performed using STATA v10.0 (Stata Corporation, TX, USA). Variables with distributions significantly different from normal were log transformed (natural log). Data are presented as mean (standard deviation) or geometric mean (95% confidence interval). Participants with a self-reported history of coronary artery disease were not excluded for analysis of fibrinogen. To determine associations between risk factors and fibrinogen, bivariate associations were assessed using linear regression. Multiple regression models with fibrinogen as the dependent variable were then calculated using backwards stepwise regression models; all established risk factors and other variables identified as significant in bivariate analyses were considered for inclusion. Goodness of fit was assessed using likelihood-ratio tests to compare nested models; a significance level of p < 0.05 was used. Associations between risk factors and fibrinogen were also assessed by grouping participants into tertiles of fibrinogen and then stratifying by gender. Factor analysis was used to examine clustering of risk factors. Factor analysis used principal components methods to reduce the information in many measured variables into a smaller set of "factors"; communalities were assumed to be one. Kaiser's criterion (eigen values > 1) was used to determine the number of factors that best described the underlying relationship among variables. The extracted factors were rotated using varimax rotation. Significant correlations were considered for variables loading ≥ 0.40.

### Ethical Approval

Ethics approval was given by the combined Human Research Ethics Committee of Northern Territory Department of Health and Community Services and Menzies School of Health Research, Darwin. This included review by both the Aboriginal Sub-Committee and the main committee.

## Results

**Figure 1:**
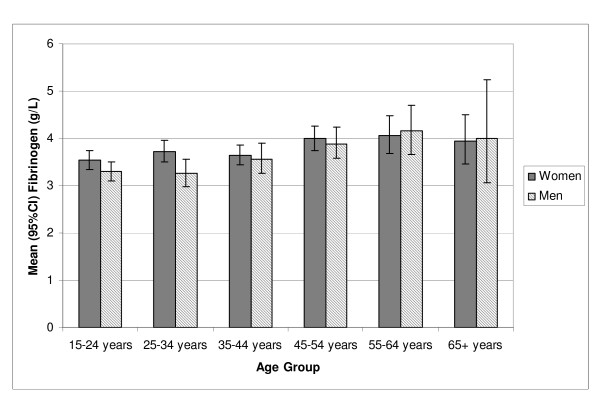
**Fibrinogen by age group and gender**.

Characteristics of participants are presented in Table 1. Fibrinogen levels for n = 915 DRUID participants generally increased with age in both genders (Figure 1). Mean (geometric) fibrinogen was 3.75 g/L for women (mean age 37 years) and 3.52 g/L for men (mean age 35 years). Across tertiles of fibrinogen, there was an increase in cardiovascular risk markers with the exception of cigarette smoking in women (Table 2). Participants with diabetes had higher mean fibrinogen than participants without diabetes (4.24 vs 3.56 g/L, p < 0.001) and the difference remained significant after adjusting for age and gender.

**Table 1:** Participant Characteristics, data are n (%)

	Female (n = 628) n (%)	Male (n = 287) n (%)
**Age Group**		
15-24 years	146 (23%)	86 (30%)
25-34 years	135 (21%)	58 (20%)
35-44 years	148 (24%)	66 (23%)
45-54 years	120 (19%)	50 (18%)
55-64 years	55 (9%)	21 (7%)
65+ years	24 (4%)	6 (2%)

**Diabetes**	121 (21%)	41 (16%)

**Cigarette Smoker**	250 (44%)	115 (43%)

**BMI Category**		
BMI < 25	212 (35%)	98 (35%)
BMI 25-29	164 (27%)	102 (36%)
BMI ≥ 30	224 (38%)	83 (29%)

**Table 2:** Cardiovascular risk markers across tertiles of fibrinogen (g/L) for urban Indigenous Australian adults in the DRUID Study.

	Fibrinogen Tertiles Females			Fibrinogen Tertiles Males		
	Low* (n = 201)	Mid* (n = 210)	High* (n = 217)	Low* (n = 119)	Mid* (n = 102)	High* (n = 66)

Age (years)	35.0 ± 13.8	36.9 ± 15.3	40.1 ± 14.3	30.4 ± 13.2	35.3 ± 13.8	41.6 ± 13.6

BMI (kg/m^2^)	26.0 ± 7.1	28.1 ± 6.8	31.8 ± 7.9	25.6 ± 5.3	28.3 ± 5.5	29.3 ± 6.1

Waist (cm)	88.3 ± 17.2	91.8 ± 16.3	100.9 ± 16.4	90.6 ± 14.1	98.3 ± 13.6	102.1 ± 16.1

Systolic BP (mmHg)	113 ± 16	114 ± 16	118 + 16	117 ± 14	122 ± 14	126 ± 17

Diastolic BP (mmHg)	71 ± 10	72 ± 10	75 ± 9	72 ± 11	75 ± 11	78 ± 10

Heart Rate (beats/min)	73 ± 11	74 ± 10	77 ± 12	70 ± 13	72 ± 12	77 ± 13

Diabetes (%)	12.2	19.0	31.0	8.2	13.5	32.8

Smoker (%)	48.4	41.5	42.3	39.8	43.8	45.9

Fibrinogen (g/L)	2.5 (2.4-2.6)	3.9 (3.88-3.96)	5.3 (5.2-5.4)	2.6 (2.5-2.8)	3.8 (3.76-3.89)	5.2 (5.1-5.4)

Total cholesterol (mmol/L)	4.8 (4.6 - 4.9)	4.9 (4.8 - 5.0)	4.9 (4.7 - 5.0)	4.8 (4.6 -5.0)	5.0 (4.8 - 5.2)	5.3 (5.1 - 5.6)

LDL Cholesterol (mmol/L)	2.8 (2.7 - 3.0)	2.9 (2.8 - 3.1)	3.0 (2.8 - 3.1)	2.9 (2.7 - 3.1)	3.0 (2.8 - 3.2)	3.3 (3.0 - 3.5)

HDL cholesterol (mmol/L)	1.2 (1.17 - 1.3)	1.15 (1.11- 1.2)	1.07(1.03- 1.1)	1.1 (1.0 - 1.2)	1.0 (0.9 - 1.0)	1.0 (0.9 - 1.0)

Triglycerides (mmol/L)	1.2 (1.1 - 1.3)	1.4 (1.3 - 1.5)	1.5 (1.4 - 1.6)	1.4 (1.3 - 1.6)	1.9 (1.6 - 2.1)	2.1 (1.8 - 2.5)

HbA1c (%)	5.3 (5.2 - 5.4)	5.6 (5.4 - 5.7)	5.9 (5.8 - 6.1)	5.2 (5.1 - 5.3)	5.6 (5.4 - 5.8)	6.2 (5.8 - 6.5)

CRP (mg/L)	1.9(1.6 - 2.3)	3.2 (2.7 - 3.7)	8.0 (7.0 - 9.2)	1.2 (1.0 - 1.4)	2.5 (2.1 - 3.0)	7.0 (5.7 - 8.6)

Homocysteine (μmol/L)	8.8 (8.4-9.2)	8.8 (8.3-9.2)	9.3 (8.9-9.8)	9.6 (9.1-10.2)	11.7 (10.7-12.7)	11.4 (10.3-12.6)

ACR (mg/mmol)	0.8 (0.7 - 0.9)	1.0 (0.8 - 1.3)	1.2 (0.9 - 1.5)	0.5 (0.4 - 0.6)	0.6 (0.5 - 0.8)	1.6 (0.9 -2.7)

Values are mean ± standard deviation or geometric mean (95% confidence interval) for continuous variables or percent for dichotomous parameters.

BMI, body mass index; BP, blood pressure; CRP, c-reactive protein; ACR, urine albumin-creatinine ratio

*Fibrinogen Tertiles: Low, 0.9-3.3; Mid, 3.4-4.4; High, 4.5-9.7 g/L

Bivariate associations (correlation coefficient, r) with fibrinogen were as follows (units are as shown in Table 2): age (0.18), waist circumference (0.29), waist-hip ratio (WHR, 0.17), body mass index (BMI, 0.30), systolic blood pressure (0.14), diastolic blood pressure (0.15), heart rate (0.16), HbA1c (0.26), total cholesterol (0.07), HDL cholesterol (-0.14), triglycerides (0.12), CRP (0.46) and urine ACR (0.17) where p < 0.001 for all except gender (p = 0.002) and total cholesterol (p = 0.008). Beta coefficients for associations with categorical variables were: female gender (0.28, p < 0.001) and alcohol abstinence (0.27, p = 0.003). Associations remained significant after adjusting for age and gender with the exception of total cholesterol. The relationship between WHR and fibrinogen was significantly stronger for men [beta coefficient (95%CI) = 4.08(2.58-5.57)] than for women [2.07(1.06-3.07)] (p for interaction term = 0.038). By contrast, the relationship between BMI and fibrinogen was stronger for women [beta coefficient (95%CI) = 0.051(0.039-0.064)] than for men [0.048(0.026-0.070)], although this was not statistically significant (p for interaction term = 0.79). There was no significant association between fibrinogen and being a current cigarette smoker (compared to former or never smoked combined).

Factors independently associated with fibrinogen on multiple regression (adjusted for age and gender) were CRP and HbA1c (Table 3). CRP alone was associated with 25.5% of the variance in fibrinogen, with an additional 1% from the combination of age, gender and HbA1c. There were no significant interactions between gender and HbA1c or CRP. Results were similar if the model was stratified by gender (data not shown). If participants with diabetes were excluded, CRP was the only variable independently associated with fibrinogen (adjusted for age and gender).

**Table 3:** Multiple linear regression analysis of fibrinogen

	Coeff.	Std. Err.	p	95% Conf Interval
				

Age (yrs)	-0.001	0.003	0.538	-0.007, 0.004

Female	0.056	0.079	0.477	-0.098, 0.210

CRP*	0.467	0.031	< 0.001	0.406, 0.529

HbA1c	.0956	0.030	0.002	0.037, 0.155

Model r^2 ^= 26.5%, n = 900

*log transformed

Results of factor analysis for fibrinogen are presented in Table 4, stratified by gender. In women, CRP and fibrinogen both clustered with the first factor, labelled "obesity"; whereas in men, CRP clustered with obesity whilst fibrinogen (with CRP) clustered with HbA1c/ACR, labelled "metabolic".

**Table 4:** Factor analysis of fibrinogen by gender*

	Factor 1: 'Obesity'		Factor 2: 'Blood Pressure'		Factor 3: 'Metabolic'		Factor 4: 'Dyslipidaemia'	
	**F**	**M**	**F**	**M**	**F**	**M**	**F**	**M**

Age	0.21	0.13	**0.64**	**0.45**	0.17	0.28	0.13	0.46

Fibrinogen	**0.52**	0.27	0.03	0.00	0.19	**0.78**	0.12	0.04

CRP	**0.75**	**0.47**	0.20	0.19	0.12	**0.65**	0.20	0.14

Homocyst	-0.02	-0.15	0.17	0.30	0.22	**0.45**	**0.48**	-0.18

Insulin	**0.66**	**0.70**	-0.10	0.02	0.34	0.18	-0.07	0.13

HDL	**-0.41**	**-0.45**	-0.02	0.14	-0.30	0.00	-0.39	**-0.67**

Trig	0.17	0.21	0.29	0.18	0.22	0.06	**0.53**	**0.73**

HbA1c	0.27	-0.05	0.34	0.24	**0.46**	**0.52**	0.25	**0.49**

BMI	**0.90**	**0.91**	0.15	0.16	-0.09	0.04	-0.11	0.09

Waist	**0.88**	**0.88**	0.25	0.24	0.01	0.14	0.01	0.13

SBP	0.17	0.20	**0.89**	**0.89**	0.05	0.05	-0.04	0.04

DBP	0.15	0.19	**0.88**	**0.90**	-0.05	0.10	0.01	0.06

ACR	-0.01	-0.25	0.24	**0.45**	**0.50**	**0.45**	0.39	0.32

Smoker	-0.12	-0.26	-0.16	0.08	-0.31	0.16	**0.74**	0.17

Heart rate	0.04	0.10	0.12	**0.41**	0.10	**0.41**	0.12	-0.18

Alcohol	-0.01	0.07	0.04	0.15	**-0.83**	-0.06	0.15	0.03

								

**% variance explained**	**20**	**18**	**15**	**16**	**10**	**13**	**10**	**11**

Significant correlations were considered for variables loading ≥ 0.40, highlighted in bold

* Five factors with eigen value > 1 were identified in both men and women, but the last of these was not readily interpretable and has not been considered further.

## Discussion

We have reported that amongst this high risk population (urban Indigenous Australians): levels of fibrinogen are similar to those previously reported for remote Aboriginal Australians but higher than those reported for Native Americans or Canadian Aboriginals; CRP was strongly associated with fibrinogen (in agreement with other studies [3,18,19]); and factor analysis revealed gender differences for clustering of fibrinogen and CRP with obesity and related metabolic factors.

Mean fibrinogen levels in The Strong Heart Study for those with diabetes compared to those without were: 3.49 vs 3.32 (mean age 59.5 vs 58.9 years) [12]; in Canadian Aboriginals of the SHARE study: mean fibrinogen was 3.69 g/L (mean age 53.1 years). The mean fibrinogen of the DRUID cohort was higher than/similar to those above at 3.68 g/L but in a strikingly younger group (mean age 36 years), although the above studies may not be directly comparable due to different methodologies used. Consistent with other reports [12,20], fibrinogen was higher in DRUID participants with diabetes compared to those without diabetes.

The Strong Heart Study of Native Americans reported that fibrinogen was a useful biomarker in significantly improving CVD risk prediction by multimarker models, whereas CRP was not so useful [12]. The authors postulate that there are differences in the prognostic properties of biomarkers when adiposity and insulin resistance are widespread in a population. Indigenous Australians are a similar high risk population to that of The Strong Heart Study with regard to high rates of insulin resistance, diabetes, obesity and premature CVD. Our finding of differences in genders between the clustering of fibrinogen and CRP on factor analysis suggests that fibrinogen is not as strongly associated with adiposity as is CRP, particularly in men, and could therefore be a more useful marker of CVD risk than CRP, as reported for Native Americans above [12]. We have previously reported a gender-differential for CRP: a stronger association with adiposity in remote Aboriginal women than men [21].

Our findings of a gender-differential on factor analysis of markers of inflammation such as CRP and fibrinogen are consistent with those of Hanley et al [22]; in that study of nondiabetic participants, adiposity measures (BMI and waist) loaded with inflammatory markers (CRP and fibrinogen) in women but with metabolic variables (insulin sensitivity) in men. However, in contrast to our findings, CRP and fibrinogen loaded together in both genders. Both that study and another previous report of factor analysis involving fibrinogen [23] reported broadly similar factors to that of our report: obesity/body mass, glucose/insulin/metabolic, inflammation, lipids and blood pressure.

It is interesting to note that CRP was strongly associated with fibrinogen on multiple regression in the current study, alone explaining 25.5% of the variance, when the full model explained 26.5% of variance in fibrinogen. The Fibrinogen Studies Collaboration reported that CRP explained 10% of the variance of fibrinogen in a large meta-analysis of 154,211 adults; cohort, age and sex explained one third of the variance and traditional CVD risks explained 7% of the variance [3]. This is consistent with the report of remote Australian Aborigines and Torres Strait Islanders, where traditional cardiovascular risks explained only 12% of the variance in fibrinogen (CRP was not assessed in that study) [10]. Although CRP and fibrinogen were closely associated, the marked difference between genders that has been reported for CRP was not apparent for fibrinogen.

Consistent with previous studies of remote Australian Aborigines, cigarette smoking was not significantly associated with fibrinogen in the DRUID cohort [10]. This is not consistent with reports from other populations [3], but could be explained by the lack of quantitative data on cigarette smoking in the current study or the high rates of cigarette smoking amongst Indigenous Australians (44% in DRUID) so that it is not a useful discriminator.

Our study has several limitations: the DRUID study is cross-sectional and composed of volunteers (two-thirds female); confounding by unmeasured factors is possible. Despite such limitations, these data represent the best currently available data for an urban Indigenous population in Australia. This is particularly important given that 73% of the total Indigenous population of Australia live in urban centres [13]. There may be similar knowledge gaps relating to other Indigenous populations internationally due to mismatches between where research is undertaken and where people actually live.

## Conclusions

Fibrinogen levels are elevated in this high-risk urban Indigenous cohort, are associated with traditional and non-traditional cardiovascular risk factors and, although closely related to CRP, may not be influenced as much by obesity as is CRP (in men). We therefore propose that fibrinogen may be a useful biomarker of CVD risk in this high-risk population and await results of longitudinal follow-up of this cohort and other longitudinal studies.

## Competing interests

The authors declare that they have no competing interests.

## Authors' contributions

LMB planned and performed analysis, drafted the manuscript. JC and KOD were DRUID study investigators, contributed to the analytical design and intellectual input to manuscript. AH contributed to the analytical design and manuscript preparation. NN contributed to manuscript preparation. All authors approved the final manuscript.

## References

[B1] Heart, stroke and vascular disease: Australian facts 2004. Cardiovascular Disease Series, No. 22 2004 Canberra: Australian Institute of Health and Welfare and National Heart Foundation of Australia

[B2] Danesh J Lewington S Thompson SG Lowe GD Collins R Kostis JB Wilson AC Folsom AR Wu K Benderly M Plasma fibrinogen level and the risk of major cardiovascular diseases and nonvascular mortality: an individual participant meta-analysis JAMA 2005 9 14 1799 1809 10.1001/jama.294.14.1799 16219884

[B3] Kaptoge S White IR Thompson SG Wood AM Lewington S Lowe GD Danesh J Associations of plasma fibrinogen levels with established cardiovascular disease risk factors, inflammatory markers, and other characteristics: individual participant meta-analysis of 154,211 adults in 31 prospective studies: the fibrinogen studies collaboration Am J Epidemiol 2007 9 8 867 879 10.1093/aje/kwm191 17785713

[B4] Sakakibara H Fujii C Naito M Plasma fibrinogen and its association with cardiovascular risk factors in apparently healthy Japanese subjects Heart Vessels 2004 9 3 144 148 10.1007/s00380-003-0753-5 15168063

[B5] Stec JJ Silbershatz H Tofler GH Matheney TH Sutherland P Lipinska I Massaro JM Wilson PF Muller JE D'Agostino RB Sr Association of fibrinogen with cardiovascular risk factors and cardiovascular disease in the Framingham Offspring Population Circulation 2000 9 14 1634 1638 11015340 10.1161/01.cir.102.14.1634

[B6] Palmieri V Celentano A Roman MJ de Simone G Best L Lewis MR Robbins DC Fabsitz RR Howard BV Devereux RB Relation of fibrinogen to cardiovascular events is independent of preclinical cardiovascular disease: the Strong Heart Study Am Heart J 2003 9 3 467 474 10.1067/mhj.2003.144 12660670

[B7] Yan RT Fernandes V Yan AT Cushman M Redheuil A Tracy R Vogel-Claussen J Bahrami H Nasir K Bluemke DA Fibrinogen and left ventricular myocardial systolic function: The Multi-Ethnic Study of Atherosclerosis (MESA) Am Heart J 2010 9 3 479 486 10.1016/j.ahj.2010.06.001 20826256 PMC2937158

[B8] Gupta AK Cornelissen G Greenway FL Dhoopati V Halberg F Johnson WD Abnormalities in circadian blood pressure variability and endothelial function: pragmatic markers for adverse cardiometabolic profiles in asymptomatic obese adults Cardiovasc Diabetol 2010 9 1 58 10.1186/1475-2840-9-58 20868493 PMC2955642

[B9] Brunner E Davey G Smith Marmot M Canner R Beksinska M O'Brien J Childhood social circumstances and psychosocial and behavioural factors as determinants of plasma fibrinogen Lancet 1996 9 9007 1008 1013 10.1016/S0140-6736(96)90147-6 8606563

[B10] Wang Z Rowley K Best J McDermott R Taylor M O'Dea K Hemostatic factors in Australian Aboriginal and Torres Strait Islander populations Metabolism 2007 9 5 629 635 10.1016/j.metabol.2006.12.009 17445537

[B11] Anand SS Yusuf S Jacobs R Davis AD Yi Q Gerstein H Montague PA Lonn E Risk factors, atherosclerosis, and cardiovascular disease among Aboriginal people in Canada: the Study of Health Assessment and Risk Evaluation in Aboriginal Peoples (SHARE-AP) The Lancet 2001 9 9288 1147 1153 10.1016/S0140-6736(01)06255-9 11597669

[B12] Kizer JR Krauser DG Rodeheffer RJ Burnett JC Jr Okin PM Roman MJ Umans JG Best LG Lee ET Devereux RB Prognostic value of multiple biomarkers in American Indians free of clinically overt cardiovascular disease (from the Strong Heart Study) Am J Cardiol 2009 9 2 247 253 10.1016/j.amjcard.2009.03.026 19576355 PMC2745719

[B13] ABS & AIHW (Australian Bureau of Statistics & Australian Institute of Health and Welfare). The Health and Welfare of Australia's Aboriginal and Torres Strait Islander Peoples 2005 Canberra

[B14] Australian Bureau of Statistics. Population Distribution, Indigenous Australians. ABS cat. no. 4705.0 2002 Canberra

[B15] Cunningham J O'Dea K Dunbar T Weeramanthri TS Zimmet P Shaw J Study Protocol - Diabetes and related conditions in urban Indigenous people in the Darwin, Australia region: aims, methods and participation in the DRUID Study BMC Public Health 2006 9 8 10.1186/1471-2458-6-8 16417641 PMC1373687

[B16] O'Dea K Cunningham J Maple-Brown L Weeramanthri T Shaw J Dunbar T Zimmet P Diabetes and cardiovascular risk factors in urban Indigenous adults: Results from the DRUID study Diabetes Res Clin Pract 2008 9 3 483 489 10.1016/j.diabres.2008.02.008 18359533

[B17] World Health Organisation. Definition, Diagnosis and Classification of Diabetes Mellitus and Its Complications 1999 Geneva: Department of Noncommunicable Disease Surveillance, WHO

[B18] Woodward M Rumley A Lowe GD Tunstall-Pedoe H C-reactive protein: associations with haematological variables, cardiovascular risk factors and prevalent cardiovascular disease Br J Haematol 2003 9 1 135 141 10.1046/j.1365-2141.2003.04387.x 12823355

[B19] Kaptoge S Di Angelantonio E Lowe G Pepys MB Thompson SG Collins R Danesh J C-reactive protein concentration and risk of coronary heart disease, stroke, and mortality: an individual participant meta-analysis Lancet 2010 9 9709 132 140 10.1016/S0140-6736(09)61717-7 20031199 PMC3162187

[B20] Jax TW Peters AJ Plehn G Schoebel FC Hemostatic risk factors in patients with coronary artery disease and type 2 diabetes - a two year follow-up of 243 patients Cardiovasc Diabetol 2009 9 48 10.1186/1475-2840-8-48 19735543 PMC2743654

[B21] Shemesh T Rowley KG Jenkins A Brimblecombe J Best JD O'Dea K Differential association of C-reactive protein with adiposity in men and women in an Aboriginal community in northeast Arnhem Land of Australia 2006 9 1 103 108 16682979 10.1038/sj.ijo.0803350

[B22] Hanley AJ Festa A D'Agostino RB Jr Wagenknecht LE Savage PJ Tracy RP Saad MF Haffner SM Metabolic and inflammation variable clusters and prediction of type 2 diabetes: factor analysis using directly measured insulin sensitivity Diabetes 2004 9 7 1773 1781 10.2337/diabetes.53.7.1773 15220201

[B23] Sakkinen PA Wahl P Cushman M Lewis MR Tracy RP Clustering of procoagulation, inflammation, and fibrinolysis variables with metabolic factors in insulin resistance syndrome Am J Epidemiol 2000 9 10 897 907 10.1093/aje/152.10.897 11092431

